# Optimal Therapy for Adults with Langerhans Cell Histiocytosis Bone Lesions

**DOI:** 10.1371/journal.pone.0043257

**Published:** 2012-08-15

**Authors:** Maria A. Cantu, Philip J. Lupo, Mrinalini Bilgi, M. John Hicks, Carl E. Allen, Kenneth L. McClain

**Affiliations:** 1 Baylor College of Medicine, Houston, Texas, United States of America; 2 Division of Epidemiology, University of Texas School of Public Health, Houston, Texas, United States of America; 3 Texas Children’s Cancer and Hematology Centers, Baylor College of Medicine, Houston, Texas, United States of America; 4 Department of Pathology, Texas Children’s Hospital, Baylor College of Medicine, Houston, Texas, United States of America; Faculté de médecine de Nantes, France

## Abstract

**Background:**

There is little data on treatment of Langerhans cell histiocytosis (LCH) in adults. Available data is on small numbers of patients with short follow-up times and no comparison of results from different treatment regimens. We analyzed the responses of adult LCH patients with bone lesions to three primary chemotherapy treatments to define the optimal one.

**Methods and Findings:**

Fifty-eight adult patients with bone lesions, either as a solitary site or as a component of multisystem disease, were analyzed for disease location and response to surgery, curettage, steroids, radiation, vinblastine/prednisone, 2-Chlorodeoxyadenosine (2-CdA), or cytosine arabinoside (ARA-C). The mean age of patients was 32 years, with equal gender distribution. Twenty-nine patients had 1 lesion; 16, 2 lesions; 5, 3 lesions; and 8 had 4 or more. Most bone lesions were in the skull, spine, or jaw. Chemotherapy, surgery, curettage, or radiation, but not steroids alone, achieved improvement or resolution of lesions in a majority of patients. Comparison of the three chemotherapy regimens revealed 84% of patients treated with vinblastine/prednisone either did not respond or relapsed within a year, whereas 59% of patients treated with 2-CdA and 21% treated with ARA-C failed. Toxicity was worse with the vinblastine/prednisone group as 75% had grade 3–4 neuropathy. Grade 3–4 cytopenias occurred in 37% of the 2-CdA -treated patients and 20% of the ARA-C-treated patients. The major limitation of this study is it is retrospective and not a clinical trial.

**Conclusions:**

ARA-C is an effective and minimally toxic treatment for LCH bone lesions in adults. In contrast, vinblastine/prednisone results in poor overall responses and excessive toxicity.

## Introduction

Langerhans cell histiocytosis is a disease of myeloid dendritic cells, lymphocytes, and macrophages mixed with eosinophils and neutrophils. [1] The accumulation of these cells causes the classic lytic bone lesions, skin rashes, lymphadenopathy, splenomegaly, and organ dysfunction of the pituitary, lung, liver, and bone marrow. [2] Bone lesions are the most frequent manifestation of LCH in children, but varying percentages of adult have been reported to have bone lesions. Islinger et al published the largest series of LCH patients with bone lesions. [3] They reviewed 541 cases of LCH evaluated at the Armed Forces Institute of Pathology over a 58 year period which included 211 adults and 330 pediatric cases. Perhaps because of the source of cases, males accounted for 75% of cases in adults. Skull lesions accounted for 28%, rib 25%, pelvis 8% and spine only 3% of patients in this series. There was no treatment or follow-up information. Arico et al published a survey of adult patients from members of the Histiocyte Society in 2003 which reported that 57% had bone involvement. [4] No information was given on the types of chemotherapy used for osseous involvement. Gotz and Fichter reviewed 58 adult LCH cases of which 50% had osseous lesions, 9 with multifocal bone lesions. No specific data for treatments applied to the bone lesions was provided [5].

Treatment options for adults have never been clarified by a clinical trial and the published literature provides minimal data on the comparative efficacy of various treatment options which include surgery/curettage, steroids, radiation, and various chemotherapy regimens. [6]–[15], summarized in Table 1. Few of these publications give long term follow-up, toxicity data, or comparison of responses to more than one regimen.

**Table 1:** Summary of Reports in the Literature on Treatment of Bone LCH Lesions in Adults.

Study (Ref. #)	No. Pts.	No./Type Bones	Therapy	Remission	Relapse	Toxicities
1 (6)	19	16	RT 7	7	7	
			Surg 11	11	11	
			Chemo 6	3	3	
2 (7)	84	60	RT Surg	78% of all pts.	9%	
3 (8)	30	30 40% skull, legs 20%, ribs 13%, spine 10%, pelvis 7% Multiple 10%	Surg Surg + RT RT RT + Chemo	CR 70% PR 13%Stable 7% Progr. 7%	30% Lower rec. rate with surgery + RT	
4 (9)	47	8	Chemo RT	Not given	Not given	
6 (11)	2	Multifocal	Vlb/pred	1	1	Neurop
7 (12)	25	25 mandible & maxilla	Surg. & RT	93%	7%	
8 (13)	30	22 spine 8 mfb	Surg. Plus Chemo 12 RT 5	87%	13%	
9 (14)	7	MS 3 MFB 4	MACOP-B	CR 71% PR 29%	43%	

Abbreviations: Ref.#: Reference number, No. number, pts: patients, RT: radiotherapy, surg: surgery, Chemo: chemotherapy, MS: multisystem LCH, MFB: multifocal bone LCH, CR: complete remission, PR: partial remission, neurop.: neuropathy, Vlb: velban, pred: prednisone; rec: recurrence.

Vinblastine and prednisone is the standard treatment for children. [16] Weitzman et al summarized the pediatric LCH-S trial in which 2-CdA was effective in 75% of relapsed patients with multifocal bone or bone and other site LCH [17].

During the early stages of the Histiocyte Society LCH-A1 trial to treat adult LCH patients with vinblastine/prednisone, we observed that many patients developed WHO grade 3–4 neuropathy and also did not appear to be responding as well as children to this regimen. It was because of these observations that we elected to stop using vinblastine/prednisone and instead chose to use ARA-C alone because of favorable results in our own clinic with this drug. Egeler et al have published results showing the efficacy of vincristine/ARA-C for treatment of new and relapsed LCH in children. [18] We chose to eliminate the vincristine and prednisone because of toxicities we observed using vinblastine and prednisone.

## Methods

### Objectives

This study was undertaken to determine the overall response of LCH bone lesions in adults to velban and prednisone, ARA-C, or 2-CdA. A secondary objective was to document the incidence of grade 3 and 4 toxicities to these therapies.

### Participants

This was a retrospective chart review of 58 consecutive LCH patients with biopsy- proven (CD1a+) bone lesions out of 124 adult LCH patients evaluated in the Baylor College of Medicine/Texas Children’s Cancer Center Histiocytosis Center from 2000 to 2011 was done to determine if there was an optimal chemotherapy regimen to treat these patients.

### Description of Procedures or Investigations Undertaken

The age, gender, location and number of lesions, other sites affected by LCH, and types of therapies used to treat each patient were recorded. The durability of the response to each therapy was determined and the response to subsequent treatments was noted. The following chemotherapy regimens were evaluated: 1) Vinblastine 6 mg/m^2^ weekly for 6 weeks then if good response every 3 weeks for 1 year coupled with prednisone 40 mg/m^2^ daily for 4 weeks then tapering over 2 weeks and if good response used as a pulse of 40 mg/m^2^ daily for 5 days every 3 weeks with vinblastine injection. 2) 2-CdA was given at 5 mg/m^2^ daily for 5 days, repeated monthly for 6 months. 3) ARA-C was infused at a dose of 100 mg/m^2^ daily for 5 days, repeated monthly for 6 months. Toxicities were graded by WHO criteria. We paid particular attention to patients who had to stop therapy because of Grade 3–4 neuropathy and cytopenias.

### Ethics

This retropective chart review was approved by the Baylor College of Medicine Institutional Review Board for use of Human Subjects in Research.

### Statistical Methods

Cases were characterized using counts and proportions for categorical variables and means and ranges for continuous variables (e.g., age at diagnosis and time to recurrence). In order to evaluate the efficacy of the three chemotherapy regimens, we determined odds ratios, 95% confidence intervals, and *P* values using unconditional logistic regression, where the referent groups was ARA-C and the outcome (failure) was defined as failure to respond or relapse in less than 1 year. Additionally, the risk of toxicity was evaluated for each regimen. All analyses were conducted using Intercooled Stata, version 10.1 (StataCorp LP, College Station, TX).

## Results

Tables 2 and 3 summarize the demographic characteristics of patients and are similar to what others have published. The small percentage of Hispanic patients seems anomalous considering the proportion of Hispanics in Houston is approximately 40%, but there may be a referral bias for patients who travel to our adult histiocytosis clinic. Likewise there were no African American adult patients. Ten percent of patients had LCH as a child and recurrent LCH bone lesions as an adult. A majority (78%) of patients had one or two bone lesions. The median length of follow-up was 8.5 years.

**Table 2:** Demographic and Clinical Characteristics of LCH Bone Subjects.

Characteristic	No. (%)
Mean age at diagnosis, years (range)	32 (18–72)
Ethnicity	
Non-Hispanic White	52 (89.7)
Hispanic	6 (10.3)
Gender	
Male	30 (51.7)
Female	28 (48.3)
Diabetes insipidis	
No	41 (70.2)
Yes	17 (29.8)
LCH as a child	
No	52 (89.6)
Yes	6 (10.4)
Pain	
No	19 (32.8)
Yes	39 (67.2)
Fatigue	
No	32 (55.2)
Yes	26 (44.8)

**Table 3:** Number of Bone Lesions Present in LCH Bone Cohort.

Number of bone lesions	Number of patients (%)
1	29 (50.0)
2	16 (27.6)
3	5 (8.6)
4	4 (6.9)
5	3 (5.2)
6	1 (1.7)

The bone lesions were in the expected locations for adults, with skull being the most frequent site (55%) followed by similar frequency of vertebrae (30%) and jaw lesions (26%), followed by pelvis (20%), ribs (18%), legs (18%), hand (13%), and shoulder (12%). Adults in our series had jaw and spine lesions more commonly than children, whose most frequent lesions after skull (>40%) are legs (13%), ribs (13%), pelvis (12%), then vertebrae (9%). [19] Forty-three percent of patients in our study had other sites of disease including skin (35%), lung (28%), pituitary (20%), oral (10%), central nervous system (CNS) mass lesions (6%), or CNS neurodegenerative syndrome findings (2%). In this cohort the incidence of diabetes insipidus was 30% which is similar to that reported in pediatric patients. [20] Many adult patients had pain with the onset of their disease and continued to have debilitating bone pain and fatigue after treatment. Durable response to therapy of LCH is a major problem, with 38% of patients having one or more recurrence (Table 4).

**Table 4:** Number of Recurrences in LCH Bone Cohort.

Number of recurrences	Number of patients (%)
0	36 (62.1)
1	13 (22.4)
2	7 (12.1)
3	1 (1.7)
4	1 (1.7)

The various types of treatments are listed in Table 5. Steroids with or without chemotherapy were used for patients with multifocal bone disease, bone plus another organ system, or patients with lesions of the orbit, sphenoid, temporal, or mastoid bones (“CNS Risk"). [21] Approximately 50% of patients treated with steroids or chemotherapy regimens showed improvement by 6 weeks of therapy. It is clear that surgery or curettage is very effective for single bone lesions as 75–83% of patients had non-active disease or were classified as better by 6 weeks after start of treatment. Patients treated with steroids alone had only a single bone lesion and most responded quickly, whereas patients with multiple bone lesions were more likely to be treated with chemotherapy +/− steroids and had slower or poor responses (Table 5). Radiation therapy for single bone lesions resulted in non-active or active disease-better responses for 81% of patients. These treatments would only be chosen for patients with skull lesions in the non- “CNS Risk sites", meaning the frontal, occipital, or parietal bones, vertebral lesions with no soft tissue component, or other long bones or ribs. Patients who had multifocal bone disease, “CNS Risk" bones (orbit, mastoid, sphenoid, or temporal bones) have been identified as areas needing treatment in children. “CNS Risk" indicates the higher frequency of developing a neurodegenerative syndrome associated with LCH in children with lesions in these specific sites. [22] There is no published data that adult patients have the same risk, but anecdotal data from our clinic suggest the association holds for adults. LCH in bone and some other site involved would be more appropriately treated with chemotherapy. Patients were treated with 4 different chemotherapy regimens: velban alone in 4 patients, velban and prednisone in 19 patients, 2-CdA in 22, and ARA-C in 24 patients. Table 6 summarizes the results of the 3 main chemotherapy regimens with response to therapy, duration of response, and toxicity. Some patients were treated with 1 regimen, some 2, and some all three before they achieved a stable remission, which is why the sum of patients from all chemotherapy types is different from the starting number of 58. We chose to classify as a “poor response" those patients whose LCH did not respond or relapsed at new bone location within a year of starting treatment. Patients treated with vinblastine/prednisone had the worst outcome with 84% meeting the poor response criteria (OR = 20.3 [compared to ARA-C], 95% CI: 4.2–98.2, *P*<0.001) and 75% had Grade 3–4 toxicity (OR = 6.0 [compared to ARA-C], 95% CI: 1.1–32.6, *P* = 0.04), which necessitated stopping treatment with vinblastine/prednisone and switching to another regimen. 2-CdA was somewhat more effective, but had a surprising failure rate of 59% in the first year. Although 5 (22%) of patients had Grade 3–4 cytopenias and therapy was delayed until their counts recovered, they did not have to be withdrawn from treatment. ARA-C treatment seemed to have a modest advantage over 2-CdA in that only 21% of patients met “poor response" criteria after 6 months of treatment. Hematologic toxicity was less frequent, 5 patients (20%).

**Table 5:** Response by Treatment Type.

Treatment		Response		
	**Non-active disease**	**Better**	**Intermediate**	**Worse**
Chemotherapy	13%	45%	19%	23%
Steroids	0	50%	50%	0
Surgery	42%	33%	8%	17%
Curettage	33%	50%	17%	0
Radiation	45%	36%	9%	10%

**Table 6:** Response by Type of Chemotherapy.

Treatment	Number of Patients	Number (%) Fail to Respond Or Relapse in 1 yr.	Odds ratio (95% CI)	*P* value	Number (%) Grade 3–4 Toxicity	Odds ratio (95% CI)	*P* value
Vinblastine/Prednisone	19	16 (84%)	20.3 (4.2–98.2)	<0.001	14 (75%)	6.0 (1.1–32.6)	0.04
2-CdA 5 mg/m2/d X5 Monthly X 6	22	13 (59%)	5.5 (1.5–20.2)	0.01	8 (37%)	1.2 (0.3–4.8)	0.83
ARA-C 100 mg/m2/d X5 Monthly X 6	24	5 (21%)	1.0 (ref)		5 (20%)	1.0 (ref)	

Table 7 summarizes the number of times each of the three most frequently used regimens was applied to patients as primary, secondary, or tertiary treatment. Over the past 5 years our practice has been to use cytarabine as the first regimen.

**Table 7:** Type of Chemotherapy by Treatment Order.

Treatment type	Treatment 1 No. (%)	Treatment 2 No. (%)	Treatment 3*No. (%)
Vinblastine/Prednisone	15 (41.7)	4 (22.2)	0 (0)
2-CdA 5 mg/m2/d X5 Monthly X 6	9 (25.0)	9 (50.0)	4 (36.4)
ARA-C 100 mg/m2/d X5 Monthly X 6	12 (33.3)	5 (27.8)	7 (63.6)

* Or higher.

## Discussion

The main findings of this study are that treatment with low dose ARA-C is the most effective and least toxic regimen when compare with velban/prednisone or 2-CdA. Given the rarity of LCH in adults, the lack of a “standard" treatment regimen, and challenges conducting a national or international trial, the data presented here represent a starting place for considering institutional pilot studies to prospectively evaluate various therapeutic regimens. Prior publications on adult LCH patients have little data on the number of relapsed patients, frequency of relapse, or comparative data on responses to different chemotherapy regimens. The percentage of recurrences in our series (38%) is comparable to results reported for children in the LCH-II trial in which treatment was for 6 months. [16] This retrospective review provides some concepts for future trials. First, these data suggest that vinblastine and prednisone may not be a good regimen for adults as 84% of patients either failed to respond or relapsed within a year of starting treatment. In general, most patients find steroid toxicity to be unacceptable and too many have extreme neuropathy from the vinblastine. 2-CdA has been an effective regimen for treating adult LCH patients for many years and is often the first one used. It has also been considered as a logical choice for salvage therapy. Until now there has been no longitudinal examination of its efficacy and toxicity. In this study 59% of adults treated with 2-CdA had a recurrence within a year or did not respond. Grade 3–4 hematologic toxicity to 2-CdA is not very frequent, but given the potential for prolonged thrombocytopenia and lymphopenia it may be better to reserve 2-CdA as a salvage treatment and consider another drug as the first choice for treating adult LCH patients. From our retrospective analysis of patients treated with a modest dose of ARA-C (100 mg/m^2^ daily for 5 days, repeated monthly for 6), there is some indication that this might be the most favorable choice for adult LCH patients who need systemic therapy. The “poor response" rate (21%) and the number of Grade 3–4 toxic events (20%) were the lowest of the three regimens reported here. We speculate that treating adult LCH patients with ARA-C for 12 months may further decrease the relapse rate.

### Limitations

Although this study was not a randomized clinical trial, it is a first step in understanding the efficacy of various treatments for adults with LCH bone lesions. Patients who were treated with more than one regimen obviously had the potential for greater hematologic toxicity. Another limitation is that relapsed patients may represent a subset whose disease is more resistant to therapy and may not be comparable to patients who were previously untreated.

These results provide a framework in which to begin discussion about clinical trials for adult LCH patients. The relatively high toxicity and suboptimal efficacy of standard pediatric therapy for LCH in adults demonstrates the need for specific therapy for adult patients. It is hoped that single or multi-institutional trials can be organized to prospectively analyze the efficacy of ARA-C and other regimens for adults with LCH.
